# Clinical factors associated with bacterial translocation in Japanese patients with type 2 diabetes: A retrospective study

**DOI:** 10.1371/journal.pone.0222598

**Published:** 2019-09-19

**Authors:** Shoko Tamaki, Akio Kanazawa, Junko Sato, Yoshifumi Tamura, Takashi Asahara, Takuya Takahashi, Satoshi Matsumoto, Yuichiro Yamashiro, Hirotaka Watada

**Affiliations:** 1 Department of Metabolism & Endocrinology, Juntendo University Graduate School of Medicine, Tokyo, Japan; 2 Sportology Center, Juntendo University Graduate School of Medicine, Tokyo, Japan; 3 Probiotics Research Laboratory, Juntendo University Graduate School of Medicine, Tokyo, Japan; 4 Yakult Central Institute, Tokyo, Japan; 5 Center for Therapeutic Innovations in Diabetes, Juntendo University Graduate School of Medicine, Tokyo, Japan; 6 Center for Identification of Diabetic Therapeutic Targets, Juntendo University Graduate School of Medicine, Tokyo, Japan; Medical University of Vienna, AUSTRIA

## Abstract

**Objective:**

To explore clinical factors associated with bacterial translocation in Japanese patients with type 2 diabetes mellitus (T2DM).

**Methods:**

The data of 118 patients with T2DM were obtained from two previous clinical studies, and were retrospectively analyzed regarding the clinical parameters associated with bacterial translocation defined as detection of bacteremia and levels of plasma lipopolysaccharide binding protein (LBP), the latter of which is thought to reflect inflammation caused by endotoxemia.

**Results:**

LBP level was not significantly different between patients with and without bacteremia. No clinical factors were significantly correlated with the detection of bacteremia. On the other hand, plasma LBP level was significantly correlated with HbA1c (r = 0.312), fasting blood glucose (r = 0.279), fasting C-peptide (r = 0.265), body mass index (r = 0.371), high-density lipoprotein cholesterol (r = -0.241), and inflammatory markers (high-sensitivity C-reactive protein, r = 0.543; and interleukin-6, r = 0.456). Multiple regression analysis identified body mass index, HbA1c, high-sensitivity C-reactive protein, and interleukin-6 as independent determinants of plasma LBP level.

**Conclusion:**

The plasma LBP level was similar in patients with and without bacteremia. While both bacteremia and LBP are theoretically associated with bacterial translocation, the detection of bacteremia was not associated with LBP level in T2DM.

## Introduction

Bacterial translocation is defined by translocation of live bacteria or their components, such as lipopolysaccharide (LPS), from the gut to the blood [1]. In particular, intake of a fat-rich diet causes changes in gut microbiota that strongly increase intestinal permeability due to malfunction of tight junction proteins such as occludin and zonula occludens-1 (ZO-1), leading to increased plasma levels of LPS, a condition known as “metabolic endotoxemia” [2]. Furthermore, increased bacterial 16S rDNA translocation to the systemic circulation is known to be associated with the subsequent onset of diabetes [3]. Thus, translocation of bacterial components to the blood, resulting in increased levels of LPS and bacterial 16S rDNA, is closely related to pathogenesis in obesity and type 2 diabetes. Indeed, in humans the induction of experimental endotoxemia using LPS was reported to induce systemic insulin resistance and elevation of inflammatory markers in adipose tissue [4]. However, due to the short half-life of LPS in the blood, it is difficult to accurately measure plasma LPS levels in clinical settings [5]. On the other hand, LPS-binding protein (LBP), a 65-kDa glycoprotein that is mainly synthesized in hepatocytes and has a long half-life in the blood [6], can bind to LPS and promotes an LPS-induced immune response via toll-like receptors in macrophages [7]. Therefore, plasma LBP may be a useful marker of inflammation caused by endotoxins [8].

In a previous case-control study, we reported that gut dysbiosis, a high detection rate of live bacteria in blood, and elevated plasma LBP levels occurred in Japanese patients with type 2 diabetes mellitus (T2DM) but not in those without T2DM [9]. As a next step, we performed a prospective randomized trial to examine whether 16-week probiotic administration could reduce translocation of live bacteria in Japanese patients with T2DM. Although the administration of the probiotic reduced the median number of live bacteria in the blood of patients with T2DM [10], no reduction in plasma LBP level was observed. These data suggest that the translocation of live bacteria is not necessarily associated with LBP level, a parameter that theoretically reflects endotoxin-induced inflammation.

Thus, in this study, using the data of the patients with T2DM who were recruited in the two abovementioned clinical studies [9, 10], we investigated the association between live bacteria count and LBP level, and investigated the relationship between these parameters and clinical factors in Japanese patients with T2DM.

## Materials and methods

### Study subjects

In this study, we performed a combined analysis of the data obtained from the two previous studies referred to above. Study 1 was a case control study conducted at Juntendo University Hospital from 2011 to 2012 [9]. A total of 50 patients with T2DM were recruited. The inclusion criteria regarding HbA1c (NGSP), age, body mass index (BMI), and medications were not applied at registration, and patients with the following conditions were excluded from the study: 1) proliferative retinopathy, 2) age ≥80 years, 3) serious liver disease (AST and/or ALT >100 IU/L) or serious kidney disease (serum creatinine >2.0 mg/dL, 4) acute heart failure, 5) malignancy, 6) inflammatory bowel disease, and 7) history of treatment with antibiotics within 3 months. Study 2 was an interventional study using a probiotic (Trial registration: University Hospital Medical Information Network: ID number 000018246) that was conducted at Juntendo University Hospital from 2015 to 2017 [10]. A total of 68 patients with T2DM before probiotic administration were recruited for this study, and the following inclusion criteria were applied at study registration: 1) age >30 but <79 years, 2) HbA1c (NGSP) ≥6.0 but <8.0%, and 3) treatment with only diet and exercise or medicines excluding α-glucosidase inhibitors. Patients were excluded from the study if any of the following conditions were diagnosed at registration: 1) serious kidney disease (serum creatinine level ≥2.0 mg/dL and/or hemodialysis), 2) serious liver disease (excluding fatty liver), 3) inflammatory bowel disease, 4) BMI <20 but ≥35 kg/m^2^, and 5) past history of digestive surgery.

### Combined analysis of study subjects

The patient data from Study 1 (n = 50) and the baseline patient data from Study 2 (n = 68) were used in the current analysis. In these patients, we used previously described methods [9, 10] to perform microbiota analysis of both feces and blood using 16S- and 23S rRNA-targeted reverse transcription quantitative PCR and qPCR, as well as biochemical assays of HbA1c, fasting blood glucose, fasting C-peptide, lipids (low-density lipoprotein cholesterol [LDL-C], high-density lipoprotein cholesterol [HDL-C], and triglycerides), fecal organic acids (short chain fatty acids [SCFAs], succinic acid, and lactic acid), and inflammatory markers (high-sensitivity C-reactive protein [Hs-CRP], LBP, tumor necrosis factor [TNF]- α, and interleukin-6 [IL-6]). In addition, we investigated clinical background factors, specifically age, BMI, and diabetes duration at registration. The protocol of this retrospective study was approved by the Human Ethics Committee of Juntendo University (number: 17–253). Written informed consent was not obtained from each participant because this was a retrospective design. Additionally, we have confirmed that all data were fully anonymized before we accessed them. Participants were given the opportunity to opt out of the research.

### Statistical analyses

All data were presented as mean ± standard deviation (SD), and analyzed using StatFlex ver. 6 (Artech Co., Osaka, Japan). Comparisons of groups with and without bacteremia were performed using the non-parametric Wilcoxon signed-rank test. The relationships between clinical variables and both plasma LBP and fecal SCFA levels were investigated by Pearson’s correlation coefficient analysis. Multiple regression analysis for predicting plasma LBP level was performed using the explanatory variables of BMI, HbA1c, fasting blood glucose, C-peptide, HDL-C, Hs-CRP, and IL-6, and a cutoff value of *P* < 0.15 was used for the stepwise procedure. For each multiple regression analysis, we calculated the value of the standard regression coefficient (Stdβ), and *P* values < 0.05 were considered statistically significant.

## Results

### Clinical characteristics of the study subjects

Table 1 shows the clinical characteristics of the study subjects. Study participants were aged 63.7 ± 9.7 years, and 43 were women. As is typical for Asian patients with T2DM, the average BMI was not very high (25.4 ± 4.3 kg/m^2^). The fasting C-peptide level (1.8 ± 0.8 ng/mL) was preserved and only 17 patients were treated with insulin. As shown in S1 Table, the detection rates of the six predominant obligate anaerobic bacterial groups (*Clostridium coccoides* group, *Clostridium leptum* subgroup, *Bacteroides fragilis* group, *Bifidobacterium*, *Atopobium* cluster, and *Prevotella*) in feces ranged from 100% to 67.8%. Additionally, the detection rates of total *Lactobacillus* and pH was 100% and those of *Clostridium perfringens* and *Pseudomonas* were relatively low (44.1% and 18.6%, respectively).

**Table 1:** Clinical characteristics of the study subjects.

n	118
Sex (male/female)	75 (63.6) / 43 (36.4)
Age (years)	63.7 ± 9.7
BMI (kg/m^2^)	25.4 ± 4.3
Duration of diabetes (years)	13.3 ± 9.4
HbA1c (%)	7.7 ± 1.3
FBG (mg/dL)	140.1 ± 36.2
C-peptide (ng/mL)	1.8 ± 0.8
LDL-C (mg/dL)	113.6 ± 31.9
HDL-C (mg/dL)	51.4 ± 14.9
TG (mg/dL)	114.6 ± 49.8
Hs-CRP (mg/dL)	0.54 ± 1.12
TNF-α (pg/mL)	1.2 ± 0.6
IL-6 (pg/mL)	2.0 ± 1.2
LBP (μg/mL)	11.2 ± 3.5
Treatment for diabetes	
No medication	22 (18.6)
Oral hypoglycemic agent therapy	78 (66.1)
SU	40 (33.9)
Metformin	52 (44.1)
Thiazolidine	8 (6.8)
DPP-4 inhibitor	56 (47.5)
Glinide	11 (9.3)
SGLT2 inhibitor	3 (2.5)
α-glucosidase inhibitor	10 (8.5)
GLP-1-receptor agonist	1 (0.8)
Insulin therapy	17 (14.4)

Data are expressed as mean ± SD. The numbers in parentheses indicate percentages (%).

BMI: Body mass index, LDL-C: low-density lipoprotein cholesterol, HDL-C: high-density lipoprotein cholesterol, FBG: fasting blood glucose, TG: triglycerides; Hs-CRP, high-sensitivity C-reactive protein, IL-6: interleukin-6, TNF-α: tumor necrosis factor-α, LBP: lipopolysaccharide-binding protein, SU: sulfonylurea, DPP-4: dipeptidyl peptidase-4, SGLT2: sodium-dependent glucose cotransporter-2, GLP-1: glucagon-like peptide-1.

### Clinical factors associated with translocation of live bacteria to the blood

Of 118 patients, 26 demonstrated bacteremia (Table 2). S2 Table shows the kinds of bacteria detected in blood and their detection rates. The total detection rate was 22.0% and the most common obligate anaerobe was the *Atopobium* cluster (9.3%), followed by the *C*. *coccoides* group (7.6%) and *C*. *leptum* subgroup (7.6%). Among facultative anaerobes, *Streptococcus* (4.2%), *Enterobacteriaceae* (0.8%), and *Staphylococcus* (0.8%) were detected in blood, but the detection rates were not as high as those of the obligate anaerobes. There were no significant differences between patients with and without bacteremia in terms of various clinical factors, inflammatory markers including LBP, and fecal parameters. Only the fasting blood glucose level was higher in patients with bacteremia compared to those without, but the difference was not significant (*P* = 0.065). In addition, the two groups did not differ significantly in the fecal bacteria count (S3 Table).

**Table 2:** Clinical characteristics of study subjects with and without bacteremia.

	No Bacteremia (n = 92)	Bacteremia (n = 26)	P
Total counts (cells/1 mL-blood)	0	5.1 ± 4.9	
Clinical factors			
Sex (male/female)	58/34	17/9	0.826
Age (years)	63.6 ± 9.7	64.0 ± 9.7	0.935
Duration of diabetes (years)	13.6 ± 9.9	12.3 ± 7.6	0.828
Body mass index (kg/m^2^)	25.4 ± 4.3	25.5 ± 4.3	0.843
Fasting blood glucose (mg/dL)	135.9 ± 31.4	155.3 ± 47.2	0.065
HbA1c (%)	7.7 ± 1.3	7.7 ± 1.3	0.896
C-peptide (ng/mL)	1.8 ± 0.8	2.0 ± 0.7	0.211
LDL-C (mg/dL)	114.1 ± 30.0	112.1 ± 38.2	0.432
HDL-C (mg/dL)	51.4 ± 15.2	51.5 ± 14.2	0.826
TG (mg/dL)	113.6 ± 47.8	118.2 ± 57.0	0.937
Inflammatory markers			
Hs-CRP (mg/dL)	0.59 ± 1.3	0.35 ± 0.3	0.843
TNF-α (pg/mL)	1.2 ± 0.6	1.3 ± 0.6	0.340
IL-6 (pg/mL)	2.0 ± 1.2	2.1 ± 1.0	0.485
LBP (μg/mL)	11.2 ± 3.5	11.2 ± 3.7	0.966
Fecal organic acids (μmol/g) and pH			
Total organic acids	94.1 ± 40.0 (100)	89.7 ± 42.9 (100)	0.600
Acetic acid	56.1 ± 24.4 (100)	55.5 ± 25.9 (100)	0.917
Propionic acid	19.4 ± 10.5 (100)	18.7 ± 10.4 (100)	0.790
Butyric acid	12.6 ± 8.5 (94.6)	11.7 ± 9.1 (88.5)	0.673
Isovaleric acid	3.7 ± 2.5 (63.0)	2.8 ± 1.5 (61.5)	0.191
Valeric acid	3.0 ± 1.7 (47.8)	2.8 ± 1.3 (57.7)	0.965
Succinic acid	2.7± 5.6 (56.5)	1.3 ± 1.0 (42.3)	0.779
Lactic acid	4.3 ± 5.3 (17.4)	3.2 ± 2.8 (11.5)	NA
Formic acid	1.2 ± 1.3 (60.8)	1.2 ± 1.1 (69.2)	0.984
pH	6.6 ± 0.7 (100)	6.6 ± 0.5 (100)	0.917

Data are expressed as mean ± SD. NA: not available, Detection rates are expressed as percentages (%). The lower limit of the measurement of fecal organic acid concentrations (μmol/g): Acetic acid 0.4, Propionic acid 0.5, Butyric acid 0.55, Isovaleric acid 0.8, Valeric acid 0.65, Succinic acid 0.08, Lactic acid 0.2, Formic acid 0.05. See Table 1 for abbreviations.

### Simple correlations between clinical parameters and plasma LBP level

**Fig 1:**
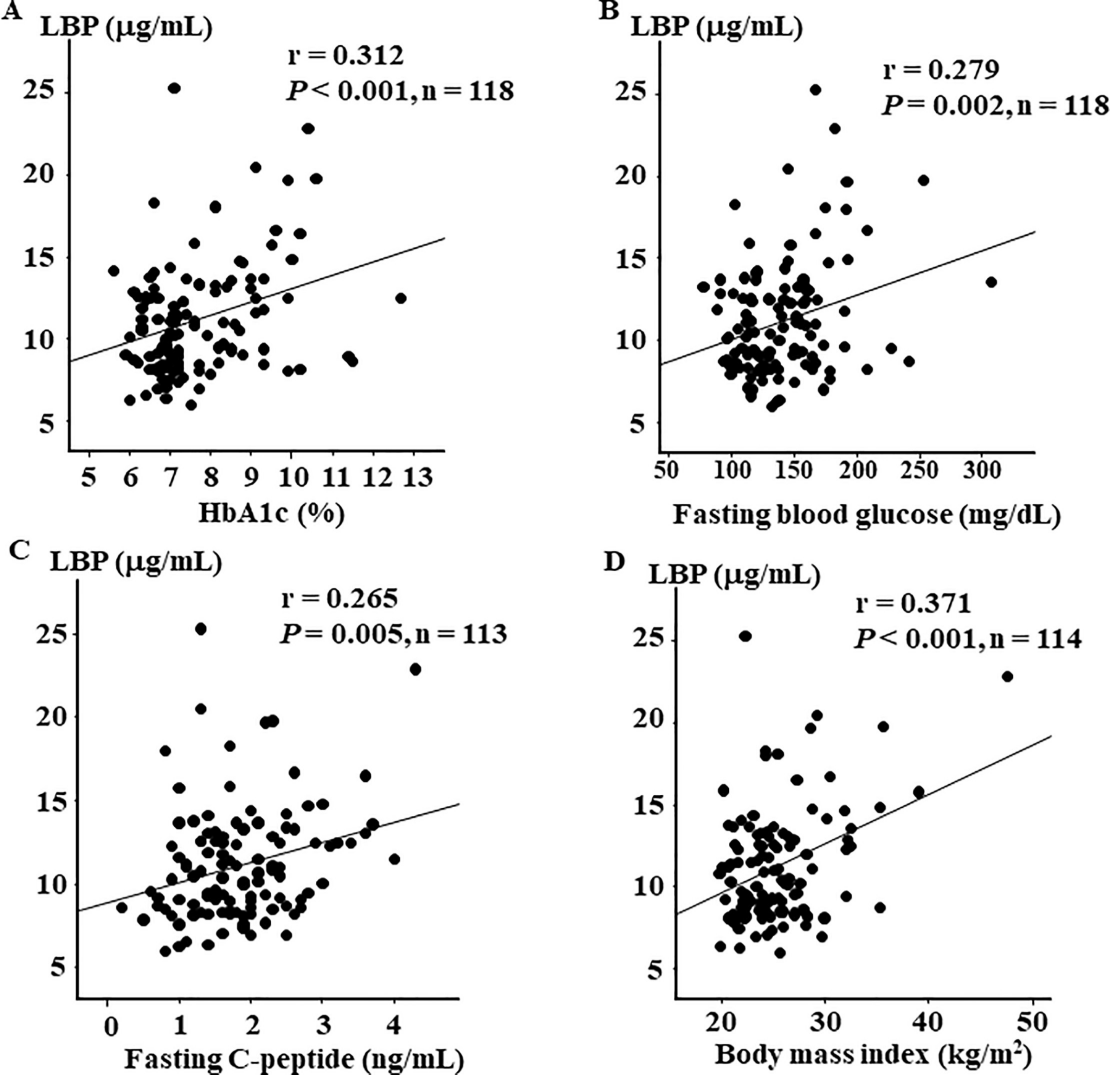
Correlations between LBP, glycemic control, and body mass index. Correlation between (A) LBP and HbA1c (r = 0.312, *P* < 0.001, n = 118), (B) LBP and fasting blood glucose (r = 0.279, *P* = 0.002, n = 118), (C) LBP and fasting C-peptide (r = 0.265, *P* = 0.005, n = 113), and (D) LBP and body mass index (r = 0.371, *P* < 0.001 n = 118).

**Fig 2:**
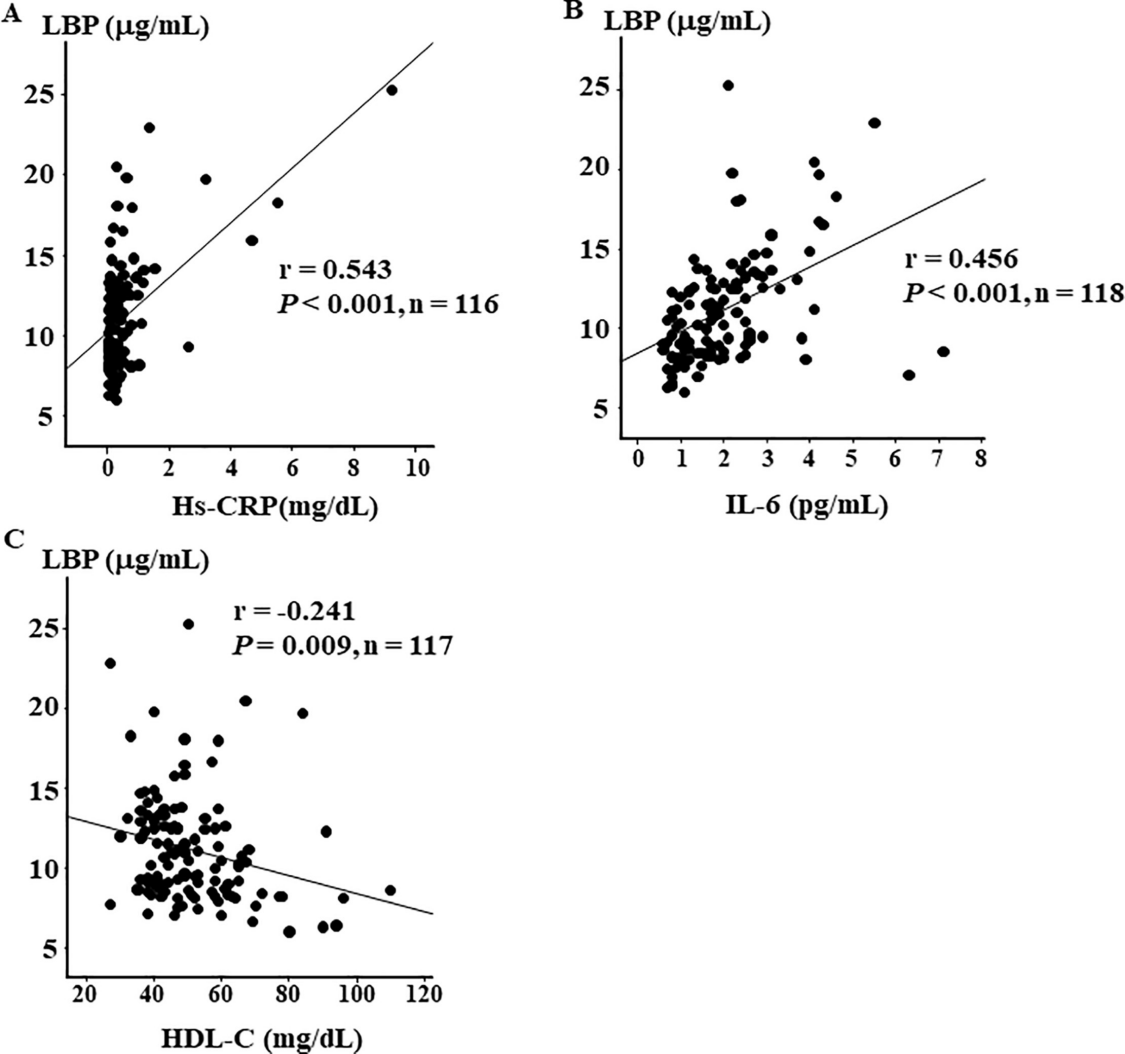
Correlations between LBP, inflammatory markers, and HDL-C. Correlation between (A) LBP and Hs-CRP (r = 0.543, *P* < 0.001, n = 116), (B) LBP and IL-6 (r = 0.456, *P* < 0.001, n = 118), and (C) LBP and HDL-C (r = - 0.241, *P* = 0.009, n = 117).

Next, to identify clinical factors associated with plasma LBP level, we calculated the correlation coefficients between plasma LBP level and other parameters. Although the correlations were weak or moderate, we found that plasma LBP level was positively associated with HbA1c (r = 0.312, *P* < 0.001, Fig 1A), fasting blood glucose (r = 0.279, *P* = 0.002, Fig 1B), fasting C-peptide levels (r = 0.265, *P* = 0.005, Fig 1C), and BMI (r = 0.371, *P* < 0.001, Fig 1D). In addition, plasma LBP level was positively associated with Hs-CRP (r = 0.543, *P* < 0.001, Fig 2A) and IL-6 (r = 0.456, *P* < 0.001, Fig 2B), and was negatively associated with HDL-C (r = -0.241, *P* = 0.009, Fig 2C). The other parameters were not associated with plasma LBP level.

### Multiple regression analysis for predicting plasma LBP level

Subsequently, multiple regression analysis was carried out to predict plasma LBP level as a dependent variable using the seven variables (BMI, HbA1c, FBG, C-peptide, HDL-C, IL-6, and Hs-CRP) that were significantly correlated with plasma LBP level in univariate analysis. As shown in Table 3, plasma LBP level was independently predicted only by BMI, Hs-CRP, and IL-6 (Model 1). Subsequent stepwise multiple regression analysis showed that plasma LBP level was independently predicted by BMI, HbA1c, Hs-CRP, and IL-6, accounting for 49.7% of the variability of the dependent variables (Model 2).

**Table 3:** Multiple regression analysis of plasma LBP with the stepwise method.

	Model 1				Model 2			
	β	Stdβ	*P*	R^2^ (R)	β	Stdβ	*P*	R ^2^(R)
BMI (kg/m^2^)	0.178	0.218	0.014	0.511 (0.737)	0.203	0.245	0.002	0.497 (0.717)
HbA1c (%)	0.337	0.128	0.147		0.447	0.168	0.030	
FBG (mg/dL)	0.008	0.079	0.340					
C-peptide (ng/mL)	-0.047	-0.010	0.904					
HDL-C (mg/dL)	-0.021	-0.089	0.249					
Hs-CRP (mg/dL)	1.552	0.514	<0.001		1.591	0.515	<0.001	
IL-6 (pg/mL)	0.654	0.223	0.004		0.647	0.216	0.004	

β: partial regression coefficient, Stdβ: standard regression coefficient

Model 1: forced entry model. Model 2: stepwise method

R^2^(R): coefficient of determination (multiple correlation coefficient)

See Table 1 for abbreviations.

### Simple correlations between clinical parameters and fecal SCFAs

**Fig 3:**
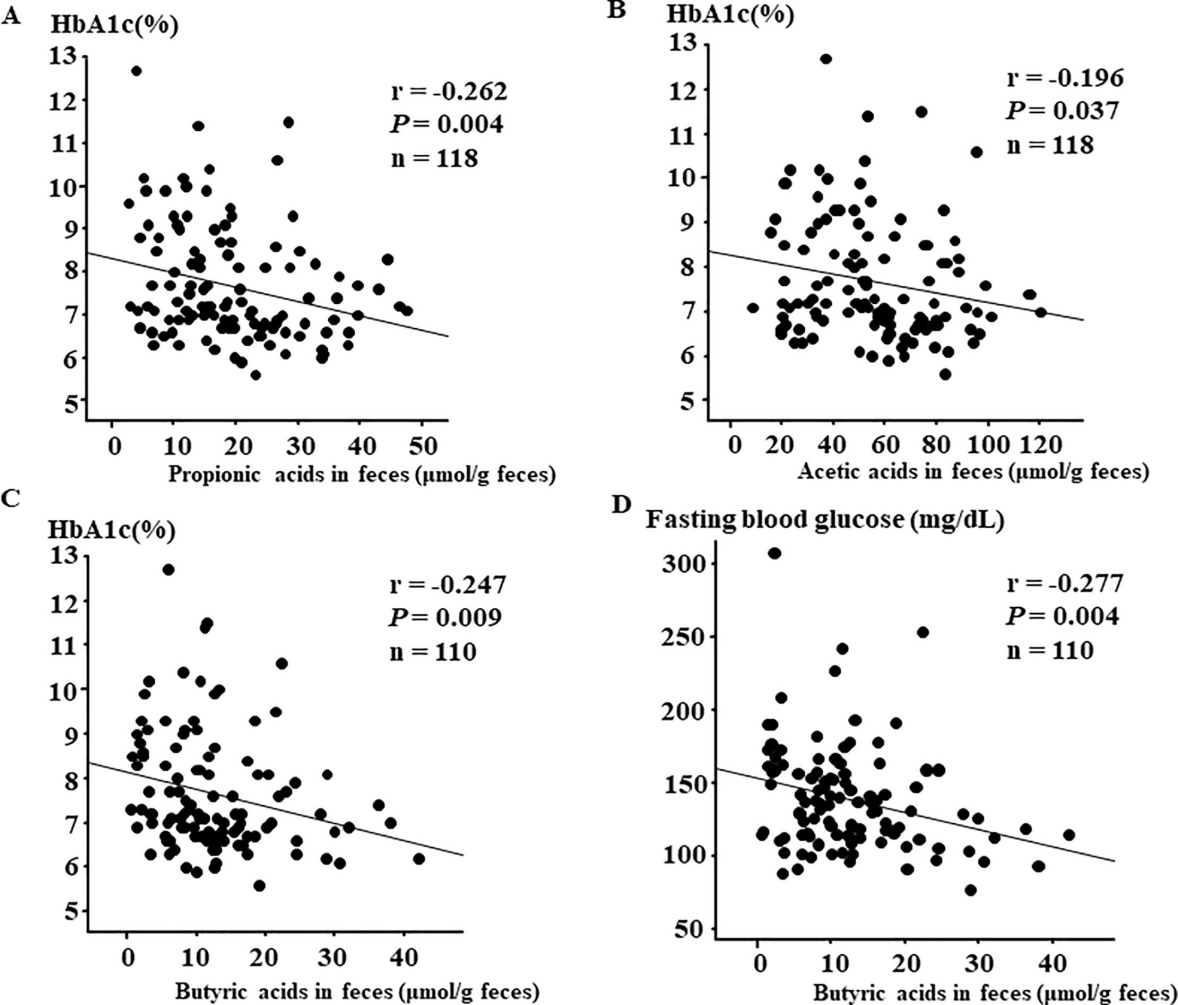
Correlations between glycemic control and organic acids in feces. Correlation between (A) HbA1c and propionic acids in feces (r = -0.262, *P* = 0.004, n = 118), (B) HbA1c and acetic acids in feces (r = -0.196, *P* = 0.037, n = 118), (C) HbA1c and butyric acids in feces (r = -0.247, *P* = 0.009, n = 110) and (D) fasting blood glucose and butyric acids in feces (r = -0.277, *P* = 0.004, n = 110).

Finally, we explored whether fecal SCFAs were associated with various clinical factors. As shown by Fig 3, weak correlations were found, and propionic acid (r = -0.262, *P* = 0.004, Fig 3A) and acetic acid (r = -0.196, *P* = 0.037, Fig 3B) were negatively correlated with HbA1c, and butyric acid was negatively correlated both with HbA1c (r = -0.247, *P* = 0.009, Fig 3C) and fasting blood glucose (r = -0.277, *P* = 0.004, Fig 3D). Regarding other parameters, although acetic acid showed a positive correlation with fasting triglyceride level (r = 0.216, *P* = 0.019), none of the SCFAs showed significant associations with BMI, age, diabetes duration, or biochemical data, including inflammatory markers.

## Discussion

In this study, we explored the clinical factors associated with bacteremia and plasma LBP, the latter of which is a marker of endotoxin-mediated inflammation. None of the clinical parameters differed between patients with and without bacteremia. On the other hand, multiple regression analysis identified BMI, HbA1c, IL-6, and Hs-CRP as independent variables associated with plasma LBP level.

In experimental mice, a high-fat diet clearly increased the translocation of bacterial DNA to mesenteric adipose tissue and the bloodstream, thereby impairing glucose metabolism, which depends on CD14 and nod-like receptors [11]. However, in this study, bacteremia in patients with T2DM was not associated with levels of inflammatory markers or with glycemic control. These results suggest that the pathophysiological mechanisms of bacteremia may differ between mice fed a high-fat diet and patients with T2DM. Thus, further studies are needed to address this issue.

In this study, we found no difference in LBP level between patients with and without bacteremia. While plasma LBP is a useful marker of inflammation caused by endotoxins, it has been reported that LBP is also an adipokine which inhibits adipogenesis, and inflammatory responses are induced in both a palmitate-dependent and LPS-dependent manner in vitro [12]. Therefore, it is possible that the pathophysiological role of LBP in patients with T2DM is not necessarily limited to LPS-mediated inflammation. In addition, the regulatory mechanism for LBP secretion in adipocytes has not yet been fully elucidated. Taking these facts into consideration, it is reasonable to assume that detection of bacteremia is not necessarily associated with LBP. It is important to develop more accurate assays for inflammation.

In this study, we found that BMI, HbA1c, IL-6, and Hs-CRP were independent variables associated with plasma LBP level. Among these, Hs-CRP was most strongly correlated with plasma LBP level because the standard regression coefficient was the highest. LBP released from hepatocytes by LPS stimulation induces IL-6 production in macrophages via toll-like receptors, which in turn activates transcriptional activity of C-reactive protein [13] and LBP itself [14] in hepatocytes. Given these close interrelations, it is not surprising that Hs-CRP and IL-6 were associated with plasma LBP level by multiple regression analysis in the present study. Furthermore, HbA1c was associated with plasma LBP level in T2DM, independently of BMI and inflammatory markers, which was consistent with the results of a previous study [15]. One possible mechanism is that bacteria and their components that are translocated from the gut to the portal vein are effectively removed from the blood via the mesenteric lymph nodes [11, 16] and Kupffer cells in liver [17]. In addition, as hyperglycemia suppresses the function of macrophages [18], impaired glycemic control might reduce the clearance of LPS from blood and consequently contribute to higher plasma LBP levels.

Regarding the relations between fecal SCFAs and clinical parameters, we found weak but significant correlations between SCFAs and HbA1c level, which was consistent with a previous report [19]. As SCFAs have been reported to stimulate GLP-1 secretion from L-cells [20, 21], fecal SCFAs in patients with T2DM might contribute to better glycemic control via the effect of incretin.

There were limitations to the present study. First, the number of subjects was small. To effectively investigate the factors contributing to bacteremia, studies with more patients may be necessary. Second, we were not able to assess the causal relationships between IL-6, Hs-CRP, and plasma LBP level because our study used a retrospective design.

In conclusion, plasma LBP level did not differ between patients with and without bacteremia. Furthermore, although we identified metabolic parameters and inflammation markers that were associated with plasma LBP level, these factors were not associated with bacteremia. While both bacteremia and LBP are theoretically associated with bacterial translocation, the detection of bacteremia was not associated with LBP in patients with T2DM in this study.

## Supporting information

S1 Table Fecal microbiota and organic acids. Bacterial counts and organic acids in feces are expressed as mean ± SD (log10 cells/g of feces and μmol/g of feces). Detection rates are expressed as percentages (%).
(DOCX)

S2 Table Bacteria detected in blood and detection rates. (DOCX)

S3 Table Fecal bacterial counts of study subjects with and without bacteremia. Bacterial counts are expressed as mean ± SD (log10 cells/g of feces). Detection rates are expressed as percentages (%).
(DOCX)

S1 Text The protocol for the study. (DOCX)
