# Supplementary material for: Clinical factors associated with bacterial translocation in Japanese patients with type 2 diabetes: A retrospective study
Source: PLoS One. 2019 Sep 19;14(9):e0222598. doi: 10.1371/journal.pone.0222598 (PMC6752875; doi:10.1371/journal.pone.0222598)
Supplement: S1 Table — Bacterial counts and organic acids in feces are expressed as mean ± SD (log10 cells/g of feces and μmol/g of feces). Detection rates are expressed as percentages (%). (DOCX) [file pone.0222598.s001.docx]

S1 Table. Fecal microbiota and organic acids

|  | **Bacterial count in feces** | **Detection rate in feces** |
| --- | --- | --- |
| n | 118 |  |
| Total bacteria | 10.3 ± 0.6 | (100.0) |
| Obligate anaerobes |  |  |
| *C. coccoides* group | 9.4 ± 0.8 | (100.0) |
| *C. leptum* subgroup | 9.5 ± 0.9 | (100.0) |
| *Bacteroides fragilis* group | 8.9 ± 0.8 | (100.0) |
| *Bifidobacterium* | 8.9 ± 1.0 | (99.9) |
| *Atopobium* cluster | 9.1 ± 0.8 | (100.0) |
| *Prevotella* | 7.7 ± 1.5 | (67.8) |
| *C. perfringens* | 4.6 ± 1.4 | (44.1) |
| Facultative anaerobes |  |  |
| Total *Lactobacillus* | 6.4 ± 1.5 | (100.0) |
| *L. gasseri* subgroup | 5.8 ± 1.6 | (87.3) |
| *L. brevis* | 4.3 ± 1.2 | (28.8) |
| *L. casei* subgroup | 4.9 ± 1.2 | (39.0) |
| *L. fermentum* | 6.1 ± 1.2 | (32.2) |
| *L. plantarum* subgroup | 4.5 ± 1.2 | (72.9) |
| *L. reuteri* subgroup | 5.3 ± 1.4 | (78.0) |
| *L. ruminis* subgroup | 5.4 ± 1.8 | (59.3) |
| *L. sakei* subgroup | 4.5 ± 1.3 | (56.8) |
| *Enterobacteriaceae* | 7.1 ± 1.2 | (95.8) |
| *Enterococcus* | 6.3 ± 1.3 | (92.4) |
| *Staphylococcus* | 4.6 ± 0.9 | (90.7) |
| Aerobes |  |  |
| *Pseudomonas* | 4.7 ± 1.4 | (18.6) |
| Total organic acids | 93.1 ± 40.5 | (100.0) |
| Acetic acid | 55.9 ± 24.6 | (100.0) |
| Propionic acid | 19.3 ± 10.4 | (100.0) |
| Butyric acid | 12.4 ± 8.6 | (93.2) |
| Isovaleric acid | 3.5 ± 2.3 | (62.7) |
| Valeric acid | 2.9 ± 1.6 | (50.0) |
| Succinic acid | 2.5 ± 5.1 | (53.4) |
| Formic acid | 1.2 ± 1.2 | (62.7) |
| Lactic acid | 4.1 ± 4.9 | (16.1) |
| pH | 6.6 ± 0.6 | (100.0) |

Bacterial counts and organic acids in feces are expressed as mean ± SD (log_10_ cells/g of feces and µmol/g of feces). Detection rates are expressed as percentages (%).
