# Supplementary material for: Clinical factors associated with bacterial translocation in Japanese patients with type 2 diabetes: A retrospective study
Source: PLoS One. 2019 Sep 19;14(9):e0222598. doi: 10.1371/journal.pone.0222598 (PMC6752875; doi:10.1371/journal.pone.0222598)
Supplement: S2 Table — (DOCX) [file pone.0222598.s002.docx]

S2 Table. Bacteria detected in blood and detection rates

|  | Detected number | Detection rate (%) |
| --- | --- | --- |
| Total bacteria | 26/118 | 22.0 |
| Obligate anaerobe |  |  |
| *C. coccoides* group | 9/118 | 7.6 |
| *Atopobium* cluster | 11/118 | 9.3 |
| *C. leptum* subgroup | 9/118 | 7.6 |
| *Bacteroides fragilis* group | 0/118 | 0 |
| *Bifidobacterium* | 0/118 | 0 |
| *Prevotella* | 0/118 | 0 |
| *C. perfringens* | 0/118 | 0 |
| Facultative anaerobe |  |  |
| *Lactobacillus* | 0/118 | 0 |
| *Streptococcus* | 5/118 | 4.2 |
| *Enterobacteriaceae* | 1/118 | 0.8 |
| *Staphylococcus* | 1/118 | 0.8 |
| *Enterococcus* | 0/118 | 0 |
| Aerobe |  |  |
| *Pseudomonas* | 0/118 | 0 |
