# Supplementary material for: Clinical factors associated with bacterial translocation in Japanese patients with type 2 diabetes: A retrospective study
Source: PLoS One. 2019 Sep 19;14(9):e0222598. doi: 10.1371/journal.pone.0222598 (PMC6752875; doi:10.1371/journal.pone.0222598)
Supplement: S3 Table — Bacterial counts are expressed as mean ± SD (log10 cells/g of feces). Detection rates are expressed as percentages (%). (DOCX) [file pone.0222598.s003.docx]

S3 Table.　Fecal bacterial counts of study subjects with and without bacteremia

|  | **No Bacteremia (n=92)** | **Bacteremia (n=26)** | **P** |
| --- | --- | --- | --- |
| Total bacteria | 10.2 ± 0.6 (100.0) | 10.4 ± 0.4 (100.0) | 0.440 |
| Obligate anaerobes |  |  |  |
| *C. coccoides* group | 9.4 ± 0.8 (100.0) | 9.6 ± 0.6 (100.0) | 0.284 |
| *C. leptum* subgroup | 9.5 ± 0.9 (100.0) | 9.6 ± 0.9 (100.0) | 0.846 |
| *Bacteroides fragilis* group | 8.9 ± 0.9 (100.0) | 9.2 ± 0.7 (100.0) | 0.135 |
| *Bifidobacterium* | 8.9 ± 1.1 (98.9) | 9.1 ± 0.7 (100.0) | 0.758 |
| *Atopobium* cluster | 9.1 ± 0.8 (100.0) | 9.2 ± 0.8 (100.0) | 0.232 |
| *Prevotella* | 7.8 ± 1.5 (68.5) | 7.4 ± 1.6 (65.4) | 0.414 |
| *C. perfringens* | 4.5 ± 1.4 (45.7) | 5.0 ± 1.5 (38.5) | 0.246 |
| Facultative anaerobes |  |  |  |
| Total *Lactobacillus* | 6.3 ± 1.5 (100.0) | 6.7 ± 1.5 (100.0) | 0.171 |
| *L. gasseri* subgroup | 5.7 ± 1.6 (87.0) | 6.0 ± 1.6 (88.5) | 0.547 |
| *L. brevis* | 4.2 ± 1.2 (29.3) | 4.7 ± 1.2 (26.9) | 0.120 |
| *L. casei* subgroup | 4.8 ± 1.1(37.0) | 5.2 ± 1.6 (46.2) | 0.548 |
| *L. fermentum* | 6.0 ± 1.2 (32.6) | 6.3 ± 1.3(30.8) | 0.543 |
| *L. plantarum* subgroup | 4.5 ± 1.1 (70.7) | 4.5 ± 1.4 (80.8) | 0.908 |
| *L. reuteri* subgroup | 5.2 ± 1.5 (76.1) | 5.4 ± 1.4 (84.6) | 0.552 |
| *L. ruminis* subgroup | 5.5 ± 1.7 (60.9) | 4.9 ± 1.9 (53.8) | 0.196 |
| *L. sakei* subgroup | 4.4 ± 1.2 (54.3) | 4.8 ± 1.6 (65.4) | 0.268 |
| *Enterobacteriaceae* | 7.0 ± 1.2 (95.7) | 7.3 ± 0.9 (96.2) | 0.271 |
| *Enterococcus* | 6.2 ± 1.3 (91.3) | 6.7 ± 1.2 (96.2) | 0.062 |
| *Staphylococcus* | 4.6 ± 0.9 (90.2) | 4.9 ± 1.0 (92.3) | 0.147 |
| Aerobes |  |  |  |
| *Pseudomonas* | 4.4 ± 1.3 (20.0) | 5.9 ± 1.1(15.4) | 0.050 |

Bacterial counts are expressed as mean ± SD (log_10_ cells/g of feces). Detection rates are expressed as percentages (%).
