# Supplementary material for: Clinical factors associated with bacterial translocation in Japanese patients with type 2 diabetes: A retrospective study
Source: PLoS One. 2019 Sep 19;14(9):e0222598. doi: 10.1371/journal.pone.0222598 (PMC6752875; doi:10.1371/journal.pone.0222598)
Supplement: S1 Text — (DOCX) [file pone.0222598.s004.docx]

**Project summary**

The data of 118 patients with type 2 diabetes were obtained from two previous clinical studies (Study 1: Sato J et al. Diabetes care 2014 and Study 2: Sato J et al. Scientific report 2017), and were retrospectively analyzed regarding the clinical parameters associated with detection of bacteremia and levels of plasma lipopolysaccharide binding protein (LBP), the latter of which is thought to reflect inflammation caused by endotoxemia.

As a result, LBP level was not significantly different between patients with and without bacteremia. No clinical factors were significantly associated with the detection of bacteremia. On the other hand, plasma LBP level was significantly associated with HbA1c (r = 0.312), fasting blood glucose (r = 0.279), fasting C-peptide (r = 0.265), body mass index (r = 0.371), high-density lipoprotein cholesterol (r = -0.241), and inflammatory markers (high-sensitivity C-reactive protein, r = 0.543; and interleukin-6, r = 0.456). Multiple regression analysis identified body mass index, HbA1c, high-sensitivity C-reactive protein, and interleukin-6 as independent determinants of plasma LBP level.

**General information**

**Protocol title**: Research Protocol for investigating clinical factors associated with bacterial translocation in Japanese patients with type 2 diabetes

**Funding**

No funding

**Name and title of the investigators**

Dr. Shoko Tamaki^1^

Associate professor Akio Kanazawa^1^,

Associate professor Junko Sato^1^,

Associate professor Yoshifumi Tamura^1,4^,

Mr. Takashi Asahara ^5,6^,

Visiting associate professor Takuya Takahashi ^5,6^,

Visiting professor Satoshi Matsumoto ^5,6^,

Professor Yuichiro Yamashiro^5^,

Professor Hirotaka Watada^1,2,3,4^

1) Department of Metabolism & Endocrinology, 2) Center for Therapeutic Innovations in Diabetes, 3) Center for Identification of Diabetic Therapeutic Targets, 4) Sportology Center, Juntendo University Graduate School of Medicine, Tokyo, 113-8421, Japan, +81-3-5802-1579

5) Probiotics Research Laboratory, Juntendo University Graduate School of Medicine, Tokyo, 113-8421, Japan, +81-3-5689-0082

6) Yakult Central Institute, Tokyo, 186-8650, Japan, +81-42-577-8960

**Rationale and background information**

In a previous case-control study, we reported that gut dysbiosis, a high detection rate of live bacteria in blood, and elevated plasma LBP levels occurred in Japanese patients with type 2 diabetes mellitus (Study 1). As a next step, we performed a prospective randomized trial to examine whether 16-week probiotic administration could reduce translocation of live bacteria in Japanese patients with type 2 diabetes mellitus (Study 2). Although the administration of the probiotic reduced the median number of live bacteria in the blood of patients with type 2 diabetes mellitus, no reduction in plasma LBP level was observed. These data suggest that the translocation of live bacteria is not necessarily associated with LBP level, a parameter that theoretically reflects endotoxin-induced inflammation.

Thus, in this study, using the data of the patients with type 2 diabetes mellitus who were recruited in the two abovementioned clinical study 1 and 2, we investigated the association between live bacteria count and LBP level, and investigated the relationship between these parameters and clinical factors in Japanese patients with type 2 diabetes mellitus.

**Study goals and objectives**

Objectives: to investigate clinical factors associated with bacterial translocation in Japanese patients with type 2 diabetes

**Study design:** A retrospective study

**Participants**

In this study, we performed a combined analysis of the data obtained from the two previous studies referred to above. Study 1 was a case control study conducted at Juntendo University Hospital from 2011 to 2012. A total of 50 patients with type 2 diabetes were recruited. The inclusion criteria regarding HbA1c (NGSP), age, body mass index (BMI), and medications were not applied at registration, and patients with the following conditions were excluded from the study: 1) proliferative retinopathy, 2) age ≥80 years, 3) serious liver disease (AST and/or ALT >100 IU/L) or serious kidney disease (serum creatinine >2.0 mg/dL, 4) acute heart failure, 5) malignancy, 6) inflammatory bowel disease, and 7) history of treatment with antibiotics within 3 months. Study 2 was an interventional study using a probiotic (Trial registration: University Hospital Medical Information Network: ID number 000018246) that was conducted at Juntendo University Hospital from 2015 to 2017. A total of 68 patients with type 2 diabetes before probiotic administration were recruited for this study, and the following inclusion criteria were applied at study registration: 1) age >30 but <79 years, 2) HbA1c (NGSP) ≥6.0 but <8.0%, and 3) treatment with only diet and exercise or medicines excluding α-glucosidase inhibitors. Patients were excluded from the study if any of the following conditions were diagnosed at registration: 1) serious kidney disease (serum creatinine level ≥2.0 mg/dL and/or hemodialysis), 2) serious liver disease (excluding fatty liver), 3) inflammatory bowel disease, 4) BMI <20 but ≥35 kg/m^2^, and 5) past history of digestive surgery.

**Methodology**

**Analysis of gut and blood microbiota used in Study 1 and 2**

**16S and 23S rRNA–targeted reverse transcription-quantitative PCR (RT-qPCR) and qPCR**

This method was previously developed by Yakult Central Institute (Tokyo, Japan). Fecal samples were weighed and then suspended in 9 volumes of RNAlater^®^ (Ambion, Austin, TX). One millilitre of blood was added to 2.0 ml of RNAprotect bacterial reagent (Qiagen, Hilden, Germany) immediately after collection. After incubation at room temperature for 10 min, the fecal samples were stored at -20°C. Blood samples were stored at -80°C, and then transported to the Yakult Central Institute. To quantify the bacteria present in the samples, we examined the gut microbiota composition and plasma levels of the gut bacteria by using the 16S and 23S rRNA–targeted RT-qPCR, Yakult Intestinal Flora-SCAN (YIF-SCAN^®^). Three serial dilutions of the extracted RNA sample were used for bacterial rRNA-targeted RT-qPCR, and the threshold cycle values in the linear range of the assay were applied to the standard curve to obtain the corresponding bacterial cell count in each nucleic acid sample. These data were then used to determine the number of bacteria per sample. The specificity of the RT-qPCR assay using group-, genus- or species-specific primers was determined as described previously (see ref. 1-4). For the enumeration of LcS in feces, Propidium monoazide (Biotium, Inc, CA, USA) treatment of fecal samples, the fecal DNA extraction and qPCR analysis was performed by the methods as described previously (see ref. 5-7).

Especially, regarding the analysis of blood microbiota (see ref. 3), high-sensitive detection was achieved by this method (detection rate: one gut bacteria / 1mL-blood). In addition, analyses of blood and fecal analyses of microbiota were performed blindly by the investigators (Takashi Asahara and Takuya Takahashi, Yakult Central Institute, Tokyo, Japan).

**Primers in this study**

| Target bacteria* | Primer | Sequence (5’ - 3’) |
| --- | --- | --- |
| *Clostridium coccoides* group | g-Ccoc-F | AAATGACGGTACCTGACTAA |
|  | g-Ccoc-R | CTTTGAGTTTCATTCTTGCGAA |
| *Clostridium leptum* subgroup | sg-Clept-F | GCACAAGCAGTGGAGT |
|  | sg-Clept-R3 | CTTCCTCCGTTTTGTCAA |
| *Bacteroides fragilis* group | g-Bfra-F2 | AYAGCCTTTCGAAAGRAAGAT |
|  | g-Bfra-R | CCAGTATCAACTGCAATTTTA |
| *Bifidobacterium* | g-Bifid-F | CTCCTGGAAACGGGTGG |
|  | g-Bifid-R | GGTGTTCTTCCCGATATCTACA |
| *Atopobium* cluster | g-Atopo-F | GGGTTGAGAGACCGACC |
|  | g-Atopo-R | CGGRGCTTCTTCTGCAGG |
| *Prevotella* | g-Prevo-F | CACRGTAAACGATGGATGCC |
|  | g-Prevo-R | GGTCGGGTTGCAGACC |
| *Akkermansia muciniphila* | AM1 | CAGCACGTGAAGGTGGGGAC |
|  | AM2 | CCTTGCGGTTGGCTTCAGAT |
| *Clostridium difficile* | Cd-lsu-F | GGGAGCTTCCCATACGGGTTG |
|  | Cd-lsu-R | TTGACTGCCTCAATGCTTGGGC |
| *Clostridium perfringens* | s-Clper-F | GGGGGTTTCAACACCTCC |
|  | ClPER-R | GCAAGGGATGTCAAGTGT |
| *Lactobacillus gasseri* subgroup | sg-Lgas-F | GATGCATAGCCGAGTTGAGAGACTGAT |
|  | sg-Lgas-R | TAAAGGCCAGTTACTACCTCTATCC |
| *Lactobacillus brevis* | s-Lbre-F | ATTTTGTTTGAAAGGTGGCTTCGG |
|  | s-Lbre-R | ACCCTTGAACAGTTACTCTCAAAGG |
| *Lactobacillus casei* subgroup | sg-Lcas-F | ACCGCATGGTTCTTGGC |
|  | sg-Lcas-R | CCGACAACAGTTACTCTGCC |
| *Lactobacillus fermentum* | LFer-1 | CCTGATTGATTTTGGTCGCCAAC |
|  | LFer-2 | ACGTATGAACAGTTACTCTCATACGT |
| *Lactobacillus fructiborans* | s-Lfru-F | TGCGCCTAATGATAGTTGA |
|  | s-Lfru-R | GATACCGTCGCGACGTGAG |
| *Lactobacillus plantarum* subgroup | sg-Lpla-F | CTCTGGTATTGATTGGTGCTTGCAT |
|  | sg-Lpla-R | GTTCGCCACTCACTCAAATGTAAA |
| *Lactobacillus reuteri* subgroup | sg-Lreu-F | GAACGCAYTGGCCCAA |
|  | sg-Lreu-R | TCCATTGTGGCCGATCAGT |
| *Lactobacillus ruminis* subgroup | sg-Lrum-F | CACCGAATGCTTGCAYTCACC |
|  | sg-Lrum-R | GCCGCGGGTCCATCCAAAA |
| *Lactobacillus sakei* subgroup | sg-Lsak-F | CATAAAACCTAMCACCGCATGG |
|  | sg-Lsak-R | TCAGTTACTATCAGATACRTTCTTCTC |
| *Enterobacteriaceae* | En-lsu-3F | TGCCGTAACTTCGGGAGAAGGCA |
|  | En-lsu-3'R | TCAAGGACCAGTGTTCAGTGTC |
| *Enterococcus* | g-Encoc-F | ATCAGAGGGGGATAACACTT |
|  | g-Encoc-R | ACTCTCATCCTTGTTCTTCTC |
| *Streptococcus* | g-Str-F | AGCTTAGAAGCAGCTATTCATTC |
|  | g-Str-R | GGATACACCTTTCGGTCTCTC |
| *Staphylococcus* | g-Staph-F | TTTGGGCTACACACGTGCTACAATGGACAA |
|  | g-Staph-R | AACAACTTTATGGGATTTGCWTGA |
| *Pseudomonas* | PSD7F | CAAAACTACTGAGCTAGAGTACG |
|  | PSD7R | TAAGATCTCAAGGATCCCAACGGCT |
| *L.casei* strain Shirota^$^ | pLcS-57F | CTCAAAGCCGTGACGGTC |
|  | pLcS-597R | ACGTGGTGCTAATAATCCTAGTG |

* Group-, genus- or species, specific primer sets were developed by using 16S rDNA sequences, except for Cd-lsu-F/R, En-lsu-3F/3’R, and g-Str-F/R, which targeted 23S rDNA.

^$^ Strain-specific primers sets for L. casei strain Shirota were developed by using the LcS-specific sequences (DDBJ/GenBank/EMBL accession number: AB246299).

**Measurement of organic acids and pH in fecal samples**

Briefly, the fecal sample was homogenized in 1 mL of distilled water, then placed in an Eppendorf tube and centrifuged at 10,000 rpm at 4°C for 10 minutes. Next, 0.9 mL of the resulting supernatant and 0.1 mL of 1.5 M perchloric acid were mixed well in a glass tube and allowed to stand at 4°C for 12 hours. The suspension was then passed through a filter with a pore size of 0.45μm (Millipore Japan, Tokyo). The sample was analyzed for organic acids using the Waters HPLC system (Waters 432 Conductivity Detector; Waters Co., Milford, MA) and pH in feces was analyzed by IQ 150 pH/Thermometer (IQ Scientific Instruments, Inc., Carlsbad, CA).

**Biochemical assays**

Blood samples were obtained after overnight fast. Serum lipids (total cholesterol, high-density lipoprotein cholesterol, low-density lipoprotein cholesterol, and triglyceride), fasting blood glucose, and HbA1c were measured with standard techniques. The plasma levels of high-sensitivity C-reactive protein, interleukin-6 and tumor necrosis factor-α were measured by latex nephelometry, chemiluminescent enzyme immunoassay and enzyme-linked immunosorbent assay in a private laboratory (SRL Laboratory, Tokyo), respectively. The plasma level of lipopolysaccharide binding protein was measured by human LBP ELISA kit (Hycult Biotech, the Netherlands).

**References regarding the analysis of gut microbiota**

1.Matsuda K, Tsuji H, Asahara T, Kado Y, Nomoto K. Sensitive quantitative detection

of commensal bacteria by rRNA-targeted reverse transcription-PCR. Applied and environmental microbiology 2007; 73:32-39

2.Matsuda K, Tsuji H, Asahara T, Matsumoto K, Takada T, Nomoto K. Establishment

of an analytical system for the human fecal microbiota, based on reverse transcription-quantitative PCR targeting of multicopy rRNA molecules. Applied and environmental microbiology 2009; 75:1961-1969

3.Sakaguchi S, Saito M, Tsuji H, Asahara T, Takata O, Fujimura J, Nagata S, Nomoto

K, Shimizu T. Bacterial rRNA-targeted reverse transcription-PCR used to identify

pathogens responsible for fever with neutropenia. J Clin Microbiol 2010; 48:1624-

1628

4.Ohigashi S, Sudo K, Kobayashi D, Takahashi O, Takahashi T, Asahara T, Nomoto K, Onodera H. Changes of the intestinal microbiota, short chain fatty acids, and fecal pH in patients with colorectal cancer. Dig Dis Sci 2013; 58:1717-1726

5.Fujimoto J, Tanigawa K, Kudo Y, Makino H, Watanabe K. Identification and quantification of viable *Bifidobacterium breve* strain Yakult in human faces by using strain-specific primers and propidium monoazide. J Appl Microbiol 2011; 110:209-217

6.Matsuki T, Watanabe K, Fujimoto J, Takada T, Tanaka R. Use of 16S rRNA gene-targeted group-specific primers for real-time PCR analysis of predominant bacteria in human feces. Applied and environmental microbiology 2004; 70:7220-7228

7.Fujimoto J, Matsuki T, Sasamoto M, Tomii Y, Watanabe K. Identification and quantification of *Lactobacillus casei* strain Shirota in human feces with strain-specific primers derived from randomly amplified polymorphic DNA. International journal of food microbiology 2008; 126:210-215

**Safety considerations**

Because this study was a retrospective design, any adverse events could not be expected.

**Statistical analysis**

All data were presented as mean ± standard deviation (SD), and analyzed using StatFlex ver. 6 (Artech Co., Osaka, Japan). Comparisons of groups with and without bacteremia were performed using the non-parametric Wilcoxon signed-rank test. The relationships between clinical variables and both plasma LBP and fecal SCFA levels were investigated by Pearson’s correlation coefficient analysis. Multiple regression analysis for predicting plasma LBP level was performed using the explanatory variables of BMI, HbA1c, fasting blood glucose, C-peptide, HDL-C, high-sensitivity C-reactive protein, and IL-6, and a cutoff value of P < 0.15 was used for the stepwise procedure. For each multiple regression analysis, we calculated the value of the standard regression coefficient (Stdβ), and P values < 0.05 were considered statistically significant.

**Expected Outcomes of the Study**

Plasma LBP levels could be related to inflammatory markers.

**Publication Policy**

Publication policy is decided by two professors (Professor Yuichiro Yamashiro and

Professor Hirotaka Watada).

**Project Management**

Shoko Tamaki collected the clinical data from the medical records of each patient. Akio Kanazawa designed the study and wrote the manuscript. Junko Sato and Yoshifumi Tamura contributed to the discussion. Takashi Asahara and Takuya Takahashi contributed to the discussion and analyzed the microbiota of the fecal and blood samples in Study 1 and 2. Satoshi Matsumoto, Yuichiro Yamashiro and Hirotaka Watada contributed to the discussion and edited the manuscript.

**Ethics**

The study protocol was approved by the Human Ethics Committee of Juntendo University.
